# Supplementary material for: Immune Checkpoint Inhibitor (ICI) Genes and Aging in Clear Cell Renal Cell Carcinoma (ccRCC): Clinical and Genomic Study
Source: Cells. 2022 Nov 17;11(22):3641. doi: 10.3390/cells11223641 (PMC9688873; doi:10.3390/cells11223641)
Supplement: Supplementary file 1 [file cells-11-03641-s001.zip › cells-1987578-supplementary.pdf]

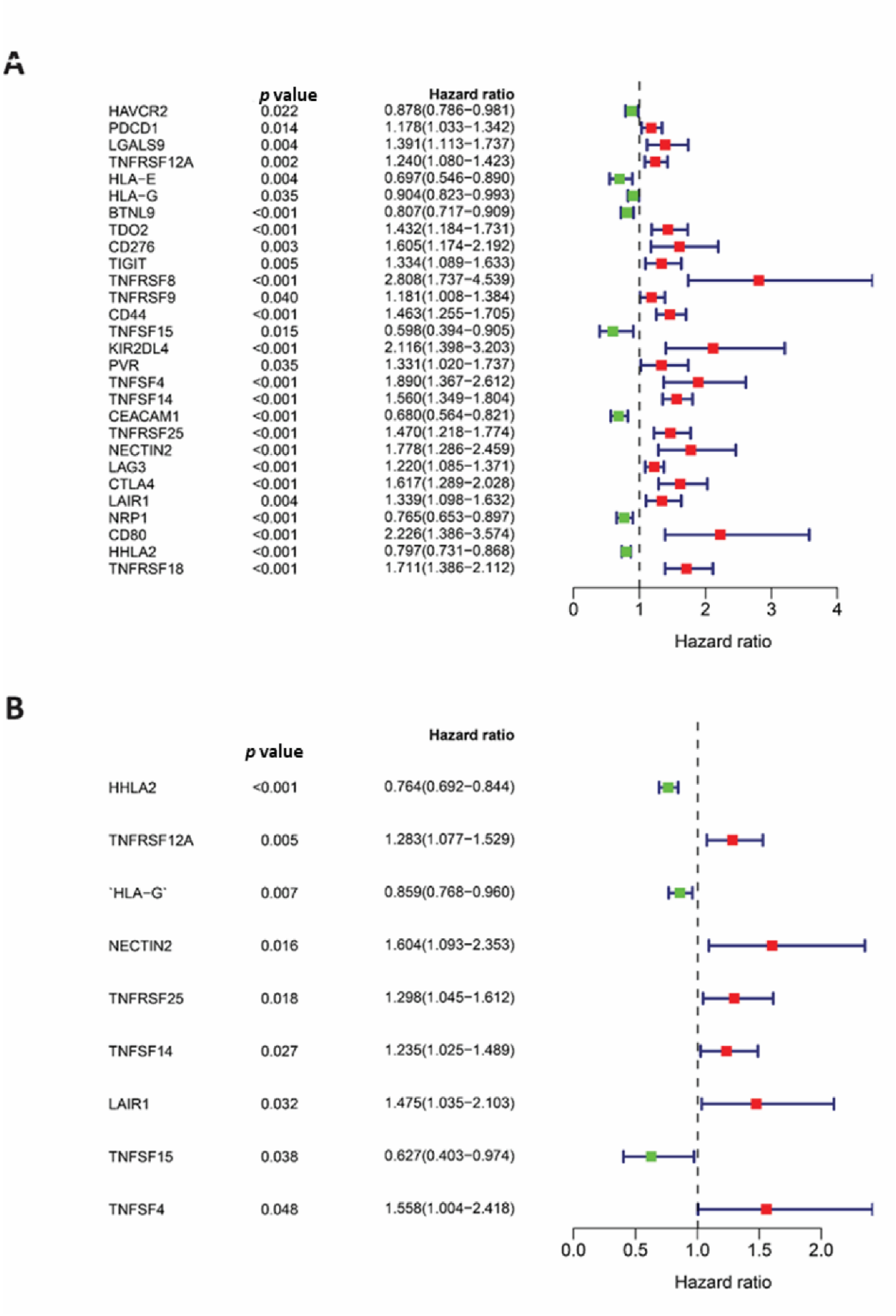

Figure S1. Construction of ICI-related gene signature. (A). Forest plot showing 28 ICIs prognostic genes for ccRCC identified by univariate with $p < 0.05$. (B). Forest plot showing 9 independent prognostic signature genes by multivariable Cox hazard regression model of ICI genes of the ccRCC cohort with $p < 0.05$.

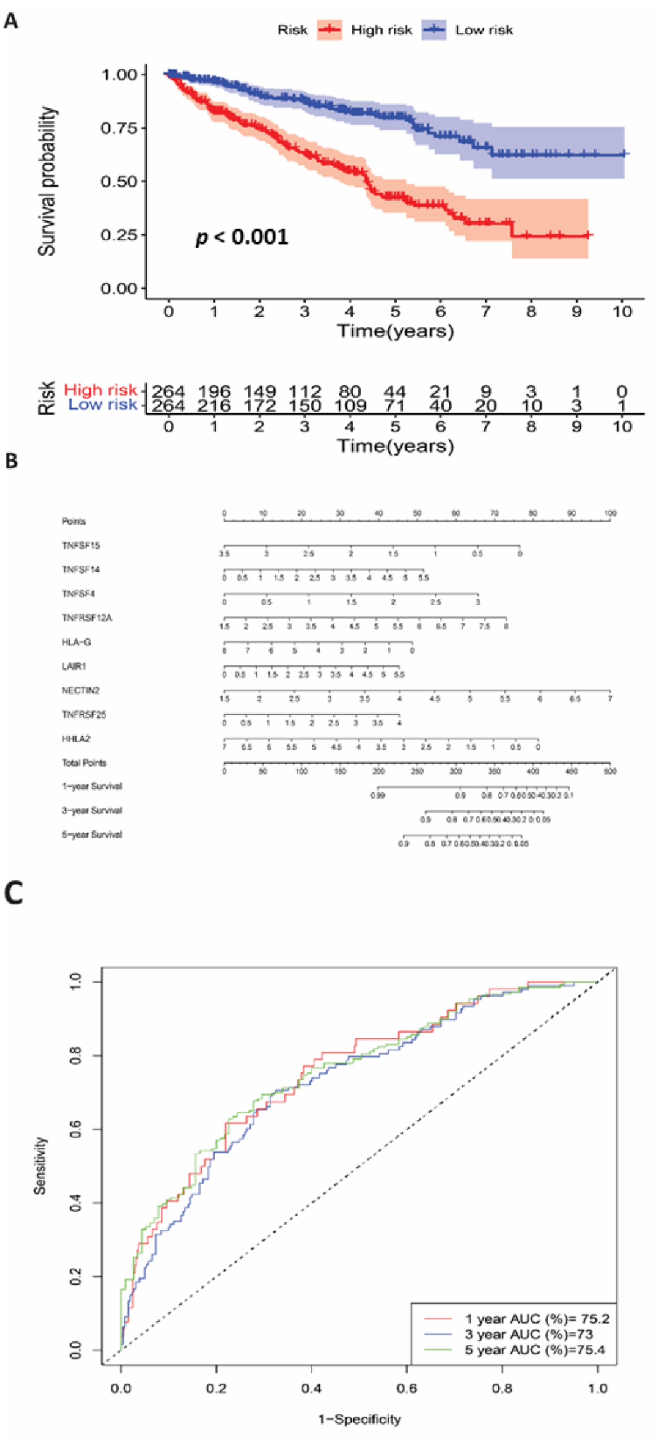

Figure S2. (A). Survival plot show significant OS differences between high and low risk group of ccRCC patients $p < 0.0001$. (B). Nomogram prediction of risk score signature genes in 1-yr, 3-yr, and 5-yr. (C). ROC curve that show predictive accuracy of risk score signature genes in 1-yr, 3-yr, and 5-yr (AUC; 75.2%,73%, and 75.4%).

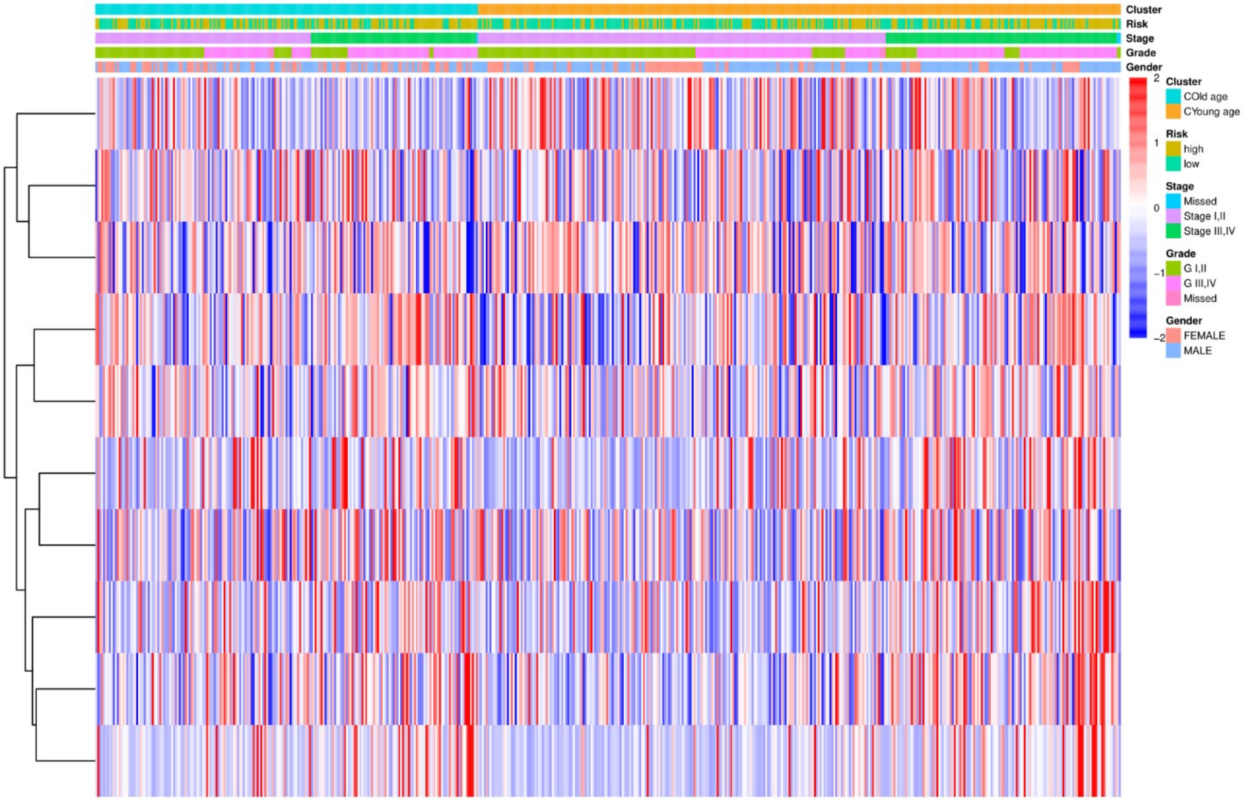

Figure S3. Heatmap showing young vs. old age group with clinicopathologic characteristics (gender, stage, and grade), risk score, and risk score signature genes.

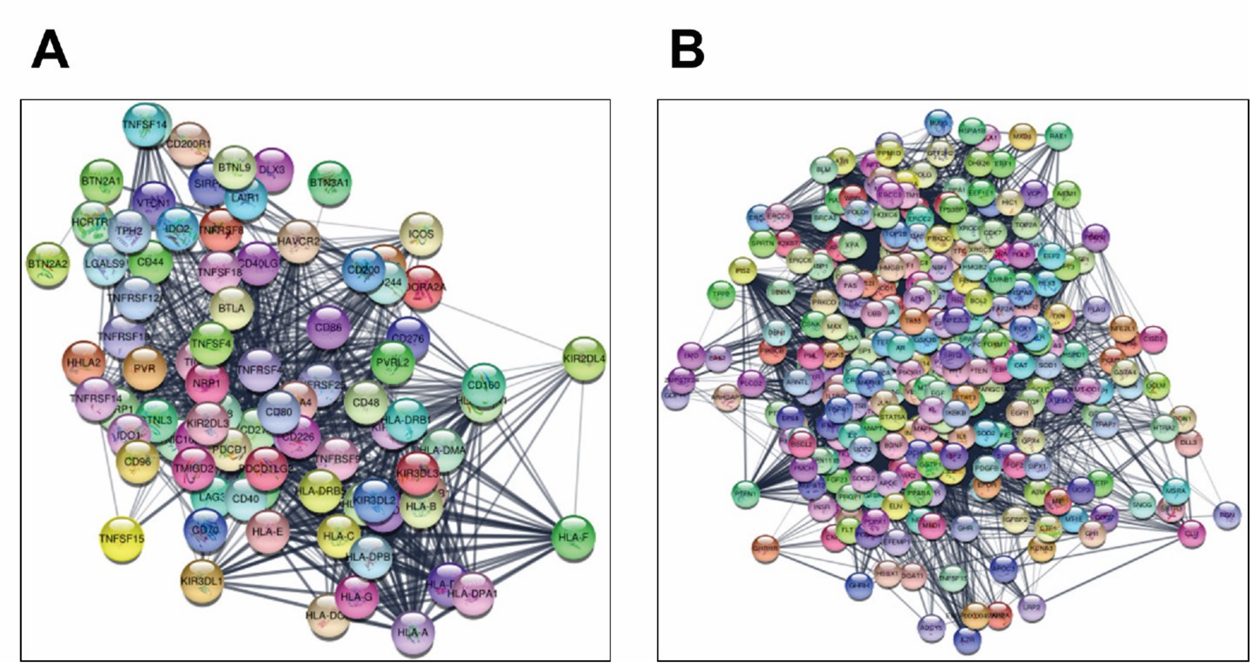

Figure S4. Protein–protein interactions (PPI) demonstrating TNFSF15 gene interplay with ICIs and aging genes. (A). ICI genes interaction with TNFSF15. (B). Aging genes list with TNFSF15.

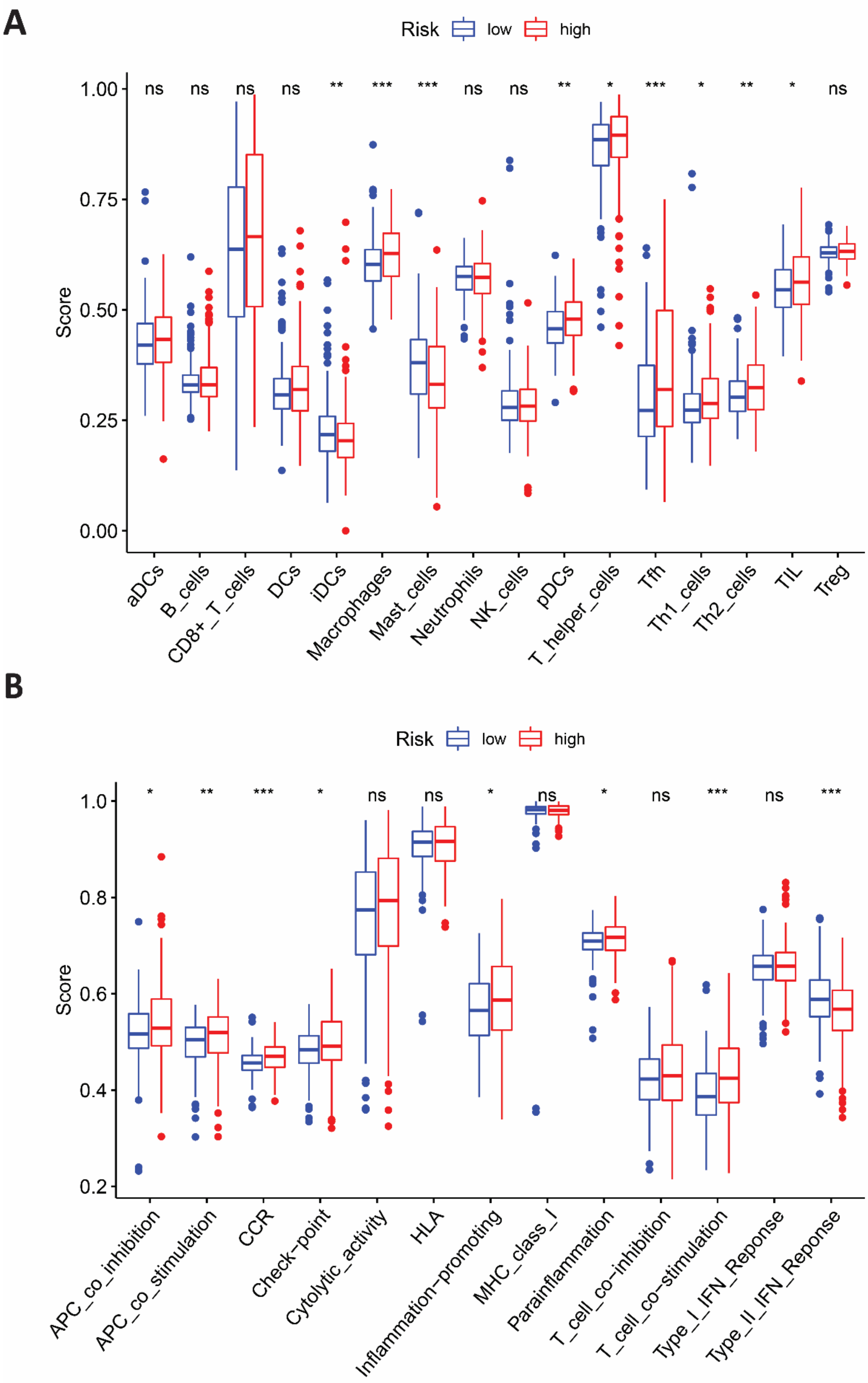
A
Risk
low
high
Score
1.00
0.75
0.50
0.25
0.00
ns ns ns ns ** *** *** ns ns ** * *** * ** * ns
aDCs
B_cells
CD8+_T_cells
DCs
iDCs
Macrophages
Mast_cells
Neutrophils
NK_cells
pDCs
T_helper_cells
Tfh
Th1_cells
Th2_cells
TIL
Treg
B
Risk
low
high
Score
1.0
0.8
0.6
0.4
0.2
* ** *** * ns ns * ns * ns *** ns ***
APC_co_inhibition
APC_co_stimulation
CCR
Check-point
Cytolytic_activity
HLA
Inflammation-promoting
MHC_class_I
Parainflammation
T_cell_co-inhibition
T_cell_co-stimulation
Type_I_IFN_Reponse
Type_II_IFN_Reponse

Figure S5. Cell infiltration and different immune function between high and low risk groups of ccRCC patients. (A). (iDCs) and mast cells were significantly higher in the low risk group $p < 0.05$. (B). Immune functional analysis. There was a significant infiltration score of type-II-IFN-response in the lower risk group than the high risk group. Checkpoint, inflammation-promoting, and T-cell-co-stimulation showed higher infiltration score in the high risk group than in the low risk group all $p < 0.05$. (ns = non-significant) * $p < 0.05$, ** $p < 0.01$, *** $p < 0.001$.

Table S1. Gene ontology and Kyoto Encyclopedia of Genes and Genomes pathways in ccRCC cohort.

GO	ONTOLOGY	Description	P value
	**BP**	Regulation of cellular senescence	0.020252379
	**BP**	positive regulation of cellular senescence	0.002568472
	**BP**	Regulation of T cell activation	3.30E-60
	**CC**	lumenal side of endoplasmic reticulum membrane	4.01E-24
	**CC**	MHC class II protein complex	5.52E-23
	**MF**	Peptide antigen binding	2.54E-24
	**MF**	Immune receptor activity	2.75E-12
	**MF**	Tumor necrosis factor receptor superfamily binding	2.16E-09
**KEGG**	**ID**	**Description**	**P value**
	**hsa04218**	Cellular senescence	0.000271365
	**hsa04658**	Th1 and Th2 cell differentiation	1.56E-10
	**hsa04060**	Cytokine-cytokine receptor interaction	1.21E-09
	**hsa05235**	PD-L1 expression and PD-1 checkpoint pathway in cancer	0.036436441

BP=biological process, CC=cellular component, MF=molecular function
